# Determinants of Early Quality-of-Life Improvement After Open Abdominal Wall Eventration Repair: A Large Single-Center Cohort Study Using the EuraHS-QoL Score

**DOI:** 10.3390/jcm15114012

**Published:** 2026-05-22

**Authors:** Laurențiu Augustus Barbu, Daniel Ioan Mihalache, Liviu Vasile, Stelian-Stefaniță Mogoantă, Tiberiu Stefăniță Țenea Cojan, Nicolae-Dragoș Mărgăritescu, Gabriel Florin Răzvan Mogoș

**Affiliations:** 1 Department of Surgery, Railway Clinical Hospital Craiova, University of Medicine and Pharmacy of Craiova, 2 Petru Rares Street, 200349 Craiova, Romania; laurentiu.barbu@umfcv.ro (L.A.B.); tiberiu.tenea@umfcv.ro (T.S.Ț.C.); gabriel.mogos@umfcv.ro (G.F.R.M.); 2 Doctoral School, “Carol Davila” University of Medicine and Pharmacy, 37 Dionisie Lupu Street, 020021 Bucharest, Romania; 3 Department of Surgery, Emergency County Ho, University of Medicine and Pharmacy of Craiova, 2 Petru Rares Street, 200349 Craiova, Romania; ssmogo@yahoo.com (S.-S.M.); dmargaritescu@yahoo.com (N.-D.M.)

**Keywords:** abdominal wall eventration, incisional hernia, quality of life, EuraHS-QoL, PROMs, open hernia repair, postoperative outcomes, abdominal wall reconstruction

## Abstract

**Background:** Abdominal wall eventration significantly affects patients’ quality of life (QoL). This study evaluated early postoperative QoL improvement after open repair and identified its determinants. **Methods:** A retrospective cohort of 1262 patients undergoing elective open abdominal wall eventration repair (2013–2022) was analyzed. QoL was assessed preoperatively and at 3 months using the EuraHS-QoL score. Multivariable linear regression identified independent predictors of QoL improvement. **Results:** EuraHS-QoL scores improved significantly from 49.6 ± 10.0 preoperatively to 16.1 ± 5.1 at 3 months (Δ = 33.5 ± 11.2; *p* < 0.001). Postoperative complications (19.0%) were associated with reduced QoL improvement and prolonged recovery. Higher baseline QoL was the strongest positive predictor (β = 0.62, *p* < 0.001), while higher body mass index, complications, longer hospital stay, and recovery time negatively influenced outcomes. Age and sex were not significant predictors. QoL improvement was comparable between primary and secondary eventration. **Conclusions:** Open eventration repair leads to significant early QoL improvement. Baseline QoL and perioperative factors are key determinants, supporting the role of patient-reported outcomes in optimizing surgical care.

## 1. Introduction

Abdominal wall eventration and incisional hernia represent a common and increasingly relevant surgical pathology, with a substantial impact on patient morbidity and healthcare systems. Traditionally considered a purely anatomical defect, ventral hernia is now recognized as a complex, multidimensional condition affecting physical function, body image, and psychosocial well-being [1,2,3].

The integrity of the abdominal wall plays a critical role in maintaining intra-abdominal pressure, trunk stability, and overall functional capacity. Disruption of this structure leads not only to structural weakness but also to pain, impaired mobility, and reduced quality of life (QoL) [4,5]. As a result, patients frequently experience limitations in daily activities, discomfort, and decreased physical performance, all of which contribute to a significant deterioration in overall health status.

Surgical repair remains the standard treatment for abdominal wall eventration, aiming to restore anatomical integrity and improve functional outcomes. Beyond traditional surgical endpoints such as recurrence and complications, increasing attention has been directed toward patient-reported outcomes, particularly QoL [6]. In this context, patient-reported outcome measures (PROMs) have emerged as essential tools for evaluating treatment effectiveness from the patient’s perspective, enabling a more comprehensive assessment of recovery and clinical success [1,7].

Among available instruments, the European Hernia Society Quality of Life (EuraHS-QoL) questionnaire is a validated disease-specific tool designed to assess pain, restriction of activity, and cosmetic discomfort following hernia repair [8,9]. Its use has allowed standardized and reproducible evaluation of postoperative outcomes and has become increasingly relevant in both clinical practice and research.

Postoperative recovery after abdominal wall reconstruction is influenced by multiple factors. In addition to surgical technique, patient-related characteristics such as body mass index, comorbidities, and baseline functional status play an important role in determining postoperative outcomes. Furthermore, recovery is closely linked to restoration of trunk muscle function and overall physical capacity, highlighting the importance of both surgical and functional aspects in patient recovery [5,10,11].

Despite growing interest in QoL assessment, most studies evaluating postoperative outcomes after hernia repair include relatively small cohorts or focus primarily on surgical techniques rather than identifying determinants of early QoL improvement. In particular, data on early postoperative QoL changes and their independent predictors in large, real-world populations remain limited.

The aim of the present study was to evaluate early postoperative quality-of-life improvement using the EuraHS-QoL score in a large single-center cohort of 1262 patients undergoing open abdominal wall eventration repair and to identify independent clinical and perioperative determinants associated with postoperative QoL improvement.

## 2. Materials and Methods

### 2.1. Study Design and Setting

This retrospective single-center cohort study included patients who underwent elective open abdominal wall eventration repair between January 2013 and December 2022 at the Emergency County Hospital of Ploiești, Romania. The study was designed to evaluate early postoperative quality-of-life improvement and to identify independent clinical and perioperative determinants associated with postoperative changes in EuraHS-QoL scores.

### 2.2. Ethical Approval

The study protocol was reviewed and approved by the Ethics Committee of the Emergency County Hospital of Ploiești, Romania. Ethical approval was granted following evaluation of the research proposal submitted to the institutional ethics board (approval decision no. 13741, 24 March 2023).

The study was conducted in accordance with the principles of the Declaration of Helsinki and institutional regulations regarding research involving human subjects. All patient data were anonymized prior to analysis and handled in accordance with the General Data Protection Regulation (GDPR). Due to the retrospective nature of the study and the use of anonymized data, the requirement for individual informed consent was waived.

### 2.3. Patient Selection

All adult patients who underwent elective open repair for abdominal wall eventration during the study period were screened for eligibility. A total of 1262 consecutive patients met the inclusion criteria and were included in the final analysis.

The inclusion criteria were as follows: age ≥ 18 years, diagnosis of abdominal wall eventration requiring elective open surgical repair, availability of complete clinical and operative records, and documented preoperative and 3-month postoperative EuraHS-QoL assessment.

The exclusion criteria were emergency procedures, incomplete medical records, absence of either preoperative or postoperative EuraHS-QoL data, and loss to follow-up before the 3-month postoperative evaluation.

### 2.4. Surgical Technique and Perioperative Management

All patients underwent elective open abdominal wall reconstruction under general anesthesia. Surgical repair was performed using a standardized retromuscular sublay technique (Rives–Stoppa procedure), which represented the preferred approach for complex abdominal wall defects during the study period.

Following median laparotomy and complete adhesiolysis, the posterior rectus sheath was opened bilaterally, and an extended retromuscular dissection was performed. Posterior sheath closure was subsequently achieved, followed by placement of a synthetic polypropylene mesh in the retromuscular plane without direct visceral contact. Restoration of the linea alba was performed through anterior fascial closure whenever feasible.

Closed-suction retromuscular drainage was routinely used in all patients. Standard perioperative management included prophylactic first- or second-generation cephalosporin administration at anesthetic induction, thromboembolic prophylaxis, postoperative analgesia, and early mobilization according to institutional protocols.

Hernia defects were classified according to the European Hernia Society (EHS) classification system based on defect width (W1 < 4 cm, W2 = 4–10 cm, W3 > 10 cm). Both primary and recurrent incisional hernias were included in the analysis.

In patients with large defects, obesity, recurrent hernias, musculofascial weakness, previous abdominal scars, or loss of abdominal wall domain, extensive retromuscular dissection and advanced abdominal wall reconstruction techniques were required to achieve tension-free closure. Component separation was selectively performed in complex cases; however, these data were not consistently available due to the retrospective design.

### 2.5. Data Collection

Clinical data were collected retrospectively from hospital electronic medical records, operative reports, discharge summaries, and follow-up documentation. The following variables were analyzed: age, sex, body mass index (BMI), type of eventration (primary or secondary), preoperative EuraHS-QoL score, postoperative EuraHS-QoL score at 3 months, length of hospital stay, recovery time, and postoperative complications. Recovery time was defined as the patient-reported interval between surgery and resumption of normal daily activities without major functional limitation.

Primary eventration was defined as a non-incisional abdominal wall defect occurring in the absence of previous abdominal surgery, whereas secondary eventration referred to an incisional hernia developing after prior abdominal surgical intervention.

Additional operative variables included hernia localization, defect size according to the European Hernia Society (EHS) classification, recurrent hernia status, operative time, mesh implantation, and perioperative surgical characteristics.

Postoperative complications included seroma, surgical site infection, and hematoma. Patients were also categorized according to the presence or absence of any postoperative complication.

### 2.6. Outcome Measures

The primary outcome of the study was early postoperative quality-of-life improvement, defined as the change in EuraHS-QoL score between the preoperative assessment and the 3-month postoperative evaluation (ΔEuraHS-QoL).

Because higher EuraHS-QoL scores indicate poorer quality of life, QoL improvement was calculated as:

ΔEuraHS-QoL = preoperative score − postoperative score.

Therefore, a positive ΔEuraHS-QoL value reflected postoperative improvement in quality of life.

### 2.7. Statistical Analysis

Statistical analysis was performed to evaluate factors associated with early postoperative quality-of-life improvement after open abdominal wall eventration repair. Continuous variables were assessed for normality using the Shapiro–Wilk test and are presented as means ± standard deviation (SD). Categorical variables are expressed as absolute frequencies and percentages.

Preoperative and postoperative EuraHS-QoL scores were compared using the paired *t*-test. Comparisons between patients with and without postoperative complications, as well as between primary and secondary eventration, were performed using the independent samples *t*-test for continuous variables and the chi-square test or Fisher’s exact test for categorical variables, as appropriate.

Correlation analysis between postoperative EuraHS-QoL score and continuous clinical variables was performed using Spearman’s correlation coefficient. Multivariable linear regression analysis was subsequently used to identify independent determinants of quality-of-life improvement, using ΔEuraHS-QoL as the dependent variable. The results of regression analyses are reported as beta coefficients (β) with 95% confidence intervals (CI).

Model performance was assessed using the adjusted R^2^ value. All statistical tests were two-tailed, and a *p*-value < 0.05 was considered statistically significant. Data were initially processed using Microsoft Excel 2016 (Microsoft Corp., Redmond, WA, USA) with XLSTAT 2022 (Addinsoft SARL, Paris, France), and statistical analyses were performed using SPSS version 27.0 (IBM Corp., Armonk, NY, USA).

## 3. Results

The study cohort included 1262 patients with a mean age of 65.4 ± 11.8 years, with a predominance of female patients (61.3%). The mean body mass index was 31.2 ± 5. kg/m^2^, and the mean preoperative EuraHS-QoL score was 49.6 ± 10.0 (Table 1).

Hernia characteristics and operative details are presented in Table 2. Most patients presented with midline hernias and medium-to-large abdominal wall defects (W2–W3), reflecting the substantial surgical complexity of the study population. Recurrent hernias were observed in 23% of cases, while retromuscular polypropylene mesh repair was consistently performed using a standardized reconstructive approach.

Early postoperative outcomes showed a substantial improvement in EuraHS-QoL scores, with a mean ΔEuraHS-QoL of 33.5 ± 11.2. The mean length of hospital stay was 7.3 ± 2.5 days, and the mean recovery time was 5.7 ± 2.0 weeks (Table 3).

A significant reduction in EuraHS-QoL scores was observed at 3 months postoperatively compared to preoperative values (mean difference −33.5, 95% CI −34.2 to −32.8; *p* < 0.001) (Table 4).

Postoperative complications occurred in 19.0% of patients, with seroma and surgical site infection being the most frequently observed events (Table 5).

Patients with postoperative complications had higher EuraHS-QoL scores at 3 months, lower QoL improvement (ΔEuraHS-QoL), and longer hospital stay and recovery time compared to those without complications, while preoperative QoL scores were similar between groups (Table 6).

Spearman correlation analysis showed weak to moderate positive correlations between EuraHS-QoL at 3 months and BMI, hospital stay, and recovery time, while age was not significantly associated with postoperative QoL (Table 7).

Multivariable linear regression analysis identified BMI, postoperative complications, hospital stay, recovery time, and baseline EuraHS-QoL score as independent factors associated with QoL improvement (ΔEuraHS-QoL). Baseline QoL showed the strongest positive association, while postoperative complications and longer recovery parameters were negatively associated with QoL improvement (Table 8).

Patients with secondary eventration were older and had higher BMI, higher preoperative and postoperative EuraHS-QoL scores, longer hospital stay, and prolonged recovery time compared to those with primary eventration. The rate of postoperative complications was also higher in the secondary eventration group, while the overall QoL improvement (ΔEuraHS-QoL) was comparable between groups (Table 9).

**Figure 1:**
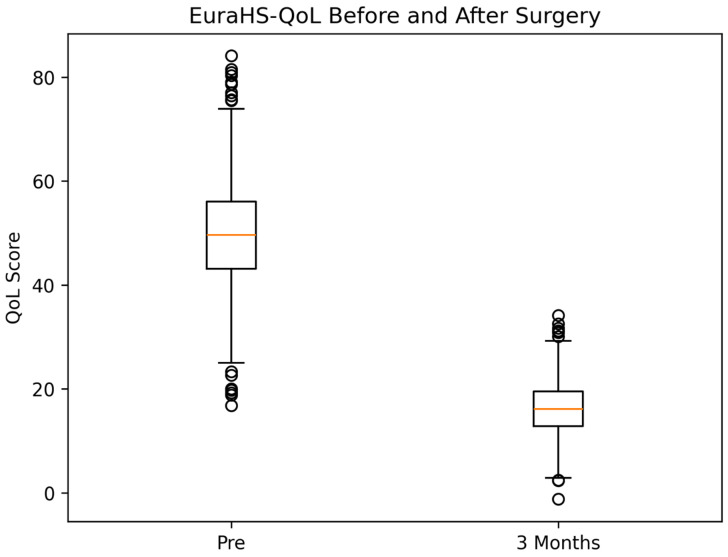
EuraHS-QoL scores preoperatively and at 3 months postoperatively.

EuraHS-QoL scores showed a marked reduction at 3 months postoperatively compared to preoperative values, indicating an overall improvement in quality of life following surgery (Figure 1).

**Figure 2:**
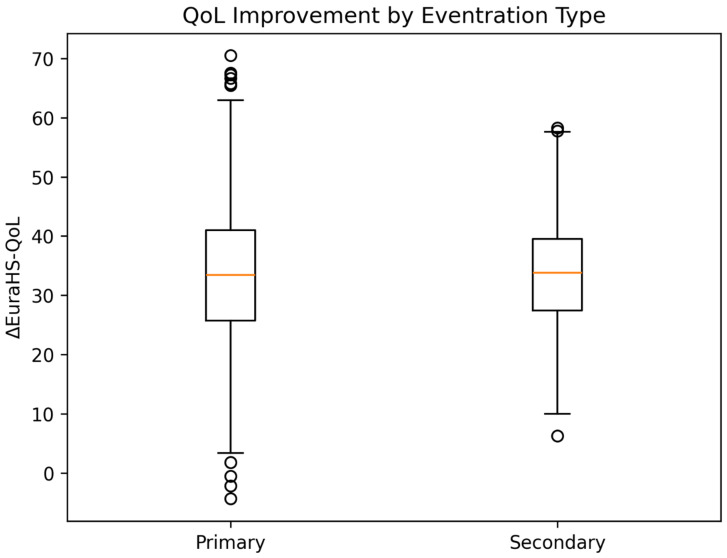
Change in EuraHS-QoL scores (ΔQoL) according to eventration type.

The improvement in EuraHS-QoL scores (ΔQoL) was similar between patients with primary and incisional eventration, with comparable distributions observed in both groups (Figure 2).

**Figure 3:**
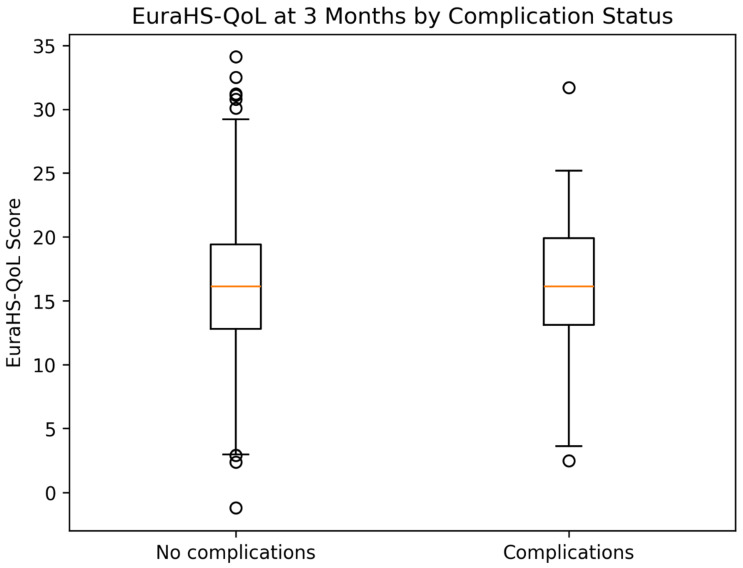
EuraHS-QoL scores at 3 months according to postoperative complications.

At 3 months postoperatively, patients with postoperative complications demonstrated significantly worse EuraHS-QoL outcomes compared to those without complications, consistent with the findings presented in Table 6 and the multivariable regression analysis (Figure 3).

**Figure 4:**
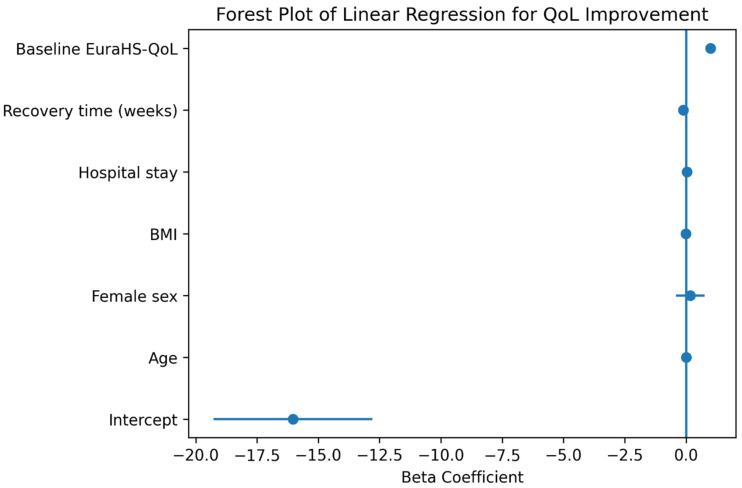
Multivariable linear regression analysis of factors associated with QoL improvement.

Multivariable linear regression analysis identified baseline EuraHS-QoL score as the strongest positive predictor of QoL improvement (ΔQoL), whereas higher body mass index, postoperative complications, prolonged hospital stay, and longer recovery time were negatively associated with postoperative QoL improvement (Figure 4).

## 4. Discussion

### 4.1. Principal Findings

The present study demonstrates a marked early improvement in quality of life following open abdominal wall eventration repair, as reflected by a substantial reduction in EuraHS-QoL scores at 3 months postoperatively. The magnitude of improvement (ΔEuraHS-QoL 33.5 ± 11.2) indicates a clinically meaningful benefit across the study population. Multivariable analysis identified baseline QoL as the strongest positive predictor of postoperative improvement, whereas higher body mass index, postoperative complications, prolonged hospital stay, and extended recovery time were independently associated with diminished QoL gains. Importantly, age and sex were not significant determinants. Although patients with secondary eventration presented with more unfavorable baseline characteristics and postoperative courses, the degree of QoL improvement remained comparable to that observed in primary cases.

### 4.2. Clinical Interpretation of QoL Improvement

These findings reinforce the concept that abdominal wall reconstruction should be evaluated not solely through traditional surgical metrics, but also through patient-centered outcomes. Ventral and incisional hernias are increasingly understood as conditions that extend beyond anatomical defects, exerting a broad impact on physical, psychological, and functional domains of health [1,12,13]. The observed postoperative improvement likely reflects a combination of pain relief, restoration of abdominal wall integrity, and improved physical performance, all of which contribute to enhanced quality of life.

The use of EuraHS-QoL as a disease-specific PROM represents a methodological strength, allowing a nuanced assessment of postoperative recovery. Unlike generic QoL instruments, EuraHS-QoL captures hernia-specific symptoms such as pain, activity restriction, and cosmetic discomfort, providing a more sensitive measure of surgical benefit [12,14]. The magnitude of change observed in this study is consistent with previous reports, supporting the clinical relevance of surgical repair even in populations with significant comorbidity burden.

### 4.3. Determinants of Postoperative QoL

The identification of baseline QoL as the strongest predictor of improvement highlights the importance of preoperative symptom burden in shaping postoperative outcomes. Patients with greater baseline impairment appear to derive disproportionate benefit from surgical intervention, suggesting that QoL metrics may assist in optimizing surgical timing and patient selection [15,16,17].

Conversely, the negative impact of postoperative complications on QoL underscores the critical role of perioperative management. Complications not only prolong recovery but also introduce additional physical and psychological stressors, ultimately diminishing patient-perceived benefit. This aligns with existing evidence demonstrating that wound-related complications significantly impair postoperative recovery trajectories and patient satisfaction [18,19,20,21].

Severe infectious complications involving deep soft tissue planes may significantly impact postoperative recovery and are associated with increased morbidity, emphasizing the importance of early recognition and aggressive management [22,23].

Body mass index emerged as an independent determinant of poorer QoL improvement. Obesity is known to affect wound healing, increase intra-abdominal pressure, and complicate surgical reconstruction, thereby influencing both short- and long-term outcomes. The present findings reinforce the importance of preoperative risk optimization, particularly in high-risk populations [24,25].

From a biological perspective, chronic inflammatory responses and tissue remodeling associated with mesh implantation may further contribute to impaired functional recovery and persistent symptoms, potentially influencing postoperative quality-of-life outcomes [26].

These findings are consistent with previous large cohort analyses that identified obesity and postoperative complications as key determinants of adverse surgical outcomes after incisional hernia repair, highlighting the critical role of patient-related and perioperative factors in shaping recovery trajectories [27].

Interestingly, age was not associated with QoL improvement in multivariable analysis. This observation is consistent with emerging evidence suggesting that biological and functional status may be more relevant than chronological age in predicting surgical outcomes [28,29]. Frailty, sarcopenia, and comorbidity burden may represent more meaningful determinants of recovery and should be considered in future analyses [30,31,32].

### 4.4. Role of Recovery Dynamics and Functional Restoration

The findings of the present study indicate that recovery-related variables, including hospital stay and recovery time, are independently associated with postoperative quality-of-life improvement.

The association between prolonged hospital stay, delayed recovery, and reduced QoL improvement suggests that recovery kinetics are central to patient-perceived outcomes. Recovery following abdominal wall reconstruction is inherently multifactorial, involving wound healing, pain control, restoration of mobility, and return to daily activities [33,34].

Importantly, incisional hernia has been shown to impair abdominal wall biomechanics, leading to reduced trunk stability, diminished muscle strength, and compromised functional capacity [5,35]. Restoration of these functions likely contributes significantly to postoperative QoL improvement.

Emerging evidence supports the role of targeted rehabilitation programs in enhancing postoperative recovery. Interventions focusing on core strengthening and functional training have been associated with improvements in muscle performance and quality of life, suggesting that structured rehabilitation may represent an underutilized strategy in hernia surgery [5,36,37]. The integration of such programs into perioperative care pathways could potentially mitigate the negative impact of prolonged recovery observed in this study.

### 4.5. Pain as a Determinant of QoL

Postoperative pain remains a key mediator of quality-of-life outcomes. Chronic pain following hernia repair is a well-recognized but variably reported complication, with prevalence estimates ranging widely due to differences in definitions and assessment tools [8,38,39].

The use of EuraHS-QoL enables standardized assessment of pain-related outcomes, facilitating comparison across studies and improving interpretability. The substantial QoL improvement observed in the present study suggests effective symptom control in the early postoperative period. However, the potential contribution of persistent or late-onset pain to long-term outcomes warrants further investigation.

The choice of a 3-month follow-up interval was intended to capture the early postoperative recovery phase, during which most patients resume daily activities and experience the initial functional and symptomatic benefits of abdominal wall reconstruction. This period is clinically relevant for evaluating short-term recovery kinetics and early patient-perceived outcomes. However, chronic postoperative pain is generally defined beyond 3–6 months after surgery, and therefore the present study cannot fully characterize persistent pain syndromes or long-term functional adaptation. Future prospective studies with 1-year or longer follow-up are required to better assess the durability of quality-of-life improvement, chronic pain, and recurrence after abdominal wall reconstruction.

### 4.6. Primary Versus Secondary Eventration

The comparative analysis between primary and secondary eventration provides additional insight into the complexity of abdominal wall pathology. Although secondary eventration was associated with higher BMI, worse baseline QoL, and increased complication rates, the overall improvement in QoL was comparable between groups. This suggests that surgical repair confers substantial benefit even in more complex clinical scenarios, albeit with a potentially more demanding recovery process.

These findings support the concept that surgical indication should not be limited by baseline severity alone, but rather guided by a comprehensive assessment of expected functional and QoL benefits [40,41].

### 4.7. Broader Clinical Context

The results of this study should be interpreted within the broader context of abdominal wall surgery, where the focus is increasingly shifting toward patient-centered care. The incorporation of PROMs into clinical practice enables a more holistic evaluation of outcomes and aligns surgical decision-making with patient expectations [42,43,44].

Furthermore, ventral hernia repair represents a significant healthcare burden, with implications for resource utilization, hospital stay, and long-term follow-up. Optimizing outcomes through risk stratification, complication prevention, and enhanced recovery protocols is therefore essential.

### 4.8. Novelty and Contribution of the Study

The present study contributes to the current literature by providing a large-scale, patient-centered evaluation of early postoperative quality-of-life outcomes following open abdominal wall eventration repair. In contrast to previous studies, which often focus on surgical techniques or long-term outcomes, this analysis specifically addresses the early postoperative period, an interval that remains insufficiently characterized despite its clinical relevance for patient recovery and satisfaction [45,46].

Importantly, the study shifts the focus from purely technical surgical outcomes toward a patient-centered framework, emphasizing the importance of functional recovery and subjective patient experience as primary indicators of treatment success. This perspective aligns with the evolving paradigm in abdominal wall surgery, where outcomes are increasingly defined by their impact on daily function and quality of life rather than solely by anatomical repair.

Furthermore, by evaluating early QoL changes in a real-world population, the study provides clinically applicable insights that may inform perioperative management strategies and support more individualized patient care. The findings reinforce the relevance of incorporating PROMs into routine clinical practice and contribute to the ongoing effort to standardize outcome assessment in hernia surgery [1,47].

Overall, the present study advances current knowledge by bridging the gap between surgical outcomes and patient experience, highlighting the importance of early postoperative recovery as a key determinant of overall treatment success.

### 4.9. Strengths and Limitations

The strengths of this study include the large cohort size, the use of a validated disease-specific QoL instrument, and the comprehensive evaluation of early postoperative outcomes. The identification of independent predictors through multivariable analysis further strengthens the clinical relevance of the findings.

However, several limitations must be acknowledged. First, the retrospective design introduces the potential for selection bias and unmeasured confounding. Second, the single-center setting may limit the generalizability of the results. Third, the assessment of quality of life was limited to the early postoperative period (3 months), and longer-term outcomes could not be evaluated due to the lack of extended follow-up, which restricts conclusions regarding the durability of QoL improvement. Consequently, the present findings primarily reflect early postoperative recovery rather than long-term functional outcomes or chronic pain trajectories.

In addition, the absence of a comparative group (e.g., minimally invasive repair or non-operative management) limits the ability to directly assess the relative impact of different treatment strategies. The study population included only surgically treated patients, which may introduce selection bias and does not allow comparison with conservatively managed cases. Detailed stratification according to mesh dimensions, fixation techniques, and component separation procedures was not consistently available for all patients due to the retrospective design. Finally, patient-reported outcomes are inherently subjective and may be influenced by individual perception, expectations, and psychosocial factors that were not captured in the present analysis. Furthermore, variables such as adherence to rehabilitation programs, postoperative pain management strategies, and psychological status were not systematically assessed and may have influenced the observed outcomes.

## 5. Conclusions

Open abdominal wall eventration repair was associated with significant early postoperative improvement in quality of life at 3 months following surgery. Baseline EuraHS-QoL score, body mass index, postoperative complications, hospital stay, and recovery time were identified as independent determinants of early postoperative benefit.

The present study provides large-scale patient-centered data regarding short-term postoperative recovery after open abdominal wall reconstruction and supports the clinical utility of EuraHS-QoL assessment in the early postoperative setting. However, further prospective studies with longer follow-up are required to evaluate the durability of QoL improvement, long-term functional outcomes, chronic pain, and recurrence rates.

## Figures and Tables

**Table 1:** Baseline Characteristics of the Study Population.

Variable	Total (N = 1262)
**Age (years), mean ± SD**	65.4 ± 11.8
**Female sex, n (%)**	774 (61.3%)
**Male sex, n (%)**	488 (38.7%)
**Body mass index (kg/m^2^), mean ± SD**	31.2 ± 5.8
**EuraHS-QoL Preoperative, mean ± SD**	49.6 ± 10.0

***Note:*** Data are presented as mean ± SD or number (%). QoL = quality of life.

**Table 2:** Hernia Characteristics and Operative Details.

Variable	Value
Midline hernia (n%)	1073 (85%)
Lateral hernia (n%)	189 (15%)
W1 defects (<4 cm), n (%)	315 (25%)
W2 defects (4–10 cm), n (%)	694 (55%)
W3 defects (>10 cm), n (%)	253 (20%)
Recurrent hernia, n (%)	290 (23%)
Mean operative time	135 ± 35 min
Retromuscular mesh placement	100%
Polypropylene mesh	100%

**Table 3:** Early Postoperative Outcomes and Quality-of-Life Improvement.

Variable	Total (N = 1262)
**EuraHS-QoL Preoperative**	49.6 ± 10.0
**EuraHS-QoL at 3 months**	16.1 ± 5.1
**ΔEuraHS-QoL (improvement)**	33.5 ± 11.2
**Length of hospital stay (days)**	7.3 ± 2.5
**Recovery time (weeks)**	5.7 ± 2.0

***Note:*** Data are presented as mean ± SD. QoL = quality of life.

**Table 4:** Statistical Comparison of Preoperative and Postoperative QoL.

Variable	Mean ± SD	Mean Difference	95% CI	*p*-Value
**EuraHS-QoL Preoperative**	49.6 ± 10.0			
**EuraHS-QoL at 3 months**	16.1 ± 5.1	−33.5	(−34.2 to −32.8)	<0.001

***Note:*** Data are presented as mean ± SD. Paired *t*-test was used. QoL = quality of life; CI = confidence interval. *p* < 0.05 was considered statistically significant.

**Table 5:** Postoperative Complications.

Complication	n (%)
**Any complication**	240 (19.0%)
**Seroma**	130 (10.3%)
**Surgical site infection (SSI)**	85 (6.7%)
**Hematoma**	44 (3.5%)

***Note:*** Data are presented as numbers (%). SSI = surgical site infection.

**Table 6:** Comparison of Postoperative Complications.

Variable	No Complications (n = 1022)	Complications (n = 240)	*p*-Value
**EuraHS-QoL Pre**	49.5 ± 10.0	50.2 ± 9.8	**0.210**
**EuraHS-QoL 3 months**	15.5 ± 4.8	18.2 ± 5.6	**<0.001**
**ΔEuraHS-QoL**	34.0 ± 11.0	32.0 ± 11.5	**0.018**
**Hospital stay (days)**	7.0 ± 2.3	8.5 ± 2.8	**<0.001**
**Recovery time (weeks)**	5.5 ± 1.8	6.8 ± 2.3	**<0.001**

***Note:*** Data are presented as mean ± SD. Continuous variables were compared using the independent samples *t*-test, and categorical variables using the chi-square test. QoL = quality of life. *p* < 0.05 was considered statistically significant.

**Table 7:** Correlation Analysis of QoL Outcomes.

Variable	EuraHS-QoL at 3 Months (r)	*p*-Value
**Age**	0.08	**0.060**
**BMI**	0.12	**0.012**
**Hospital stay**	0.34	**<0.001**
**Recovery time**	0.41	**<0.001**

***Note:*** r = Spearman correlation coefficient; QoL = quality of life; BMI = body mass index. *p* < 0.05 was considered statistically significant.

**Table 8:** Multivariable linear regression analysis of factors associated with QoL improvement (ΔEuraHS-QoL).

Variable	β Coefficient	95% CI	*p*-Value
**Age**	−0.04	(−0.08 to 0.01)	**0.120**
**Female sex**	0.85	(−0.45 to 2.15)	**0.200**
**BMI**	−0.21	(−0.35 to −0.07)	**0.003**
**Postoperative complications**	−2.10	(−3.30 to −0.90)	**<0.001**
**Hospital stay**	−0.55	(−0.72 to −0.38)	**<0.001**
**Recovery time**	−0.90	(−1.15 to −0.65)	**<0.001**
**Baseline EuraHS-QoL**	0.62	(0.55 to 0.69)	**<0.001**

***Note:*** Model = linear regression; adjusted R^2^ = 0.42. β = regression coefficient; CI = confidence interval; QoL = quality of life; BMI = body mass index. *p* < 0.05 was considered statistically significant.

**Table 9:** Comparison Between Primary and Secondary Eventration.

Variable	Primary Eventration (n = 880)	Secondary Eventration (n = 382)	*p*-Value
**Age (years)**	64.8 ± 11.5	66.7 ± 12.3	**0.012**
**Female sex, n (%)**	525 (59.7%)	249 (65.2%)	**0.080**
**BMI (kg/m^2^)**	30.9 ± 5.0	31.9 ± 5.4	**<0.001**
**EuraHS-QoL Pre**	48.8 ± 9.8	51.2 ± 10.3	**<0.001**
**EuraHS-QoL 3 months**	15.6 ± 4.9	17.2 ± 5.4	**<0.001**
**ΔEuraHS-QoL**	33.2 ± 11.0	34.0 ± 11.5	**0.210**
**Hospital stay (days)**	7.0 ± 2.3	8.0 ± 2.7	**<0.001**
**Recovery time (weeks)**	5.4 ± 1.9	6.3 ± 2.2	**<0.001**
**Any complication, n (%)**	150 (17.0%)	90 (23.5%)	**0.006**

***Note:*** Data are presented as mean ± SD or number (%). *p* < 0.05 was considered statistically significant. Abbreviations: BMI = body mass index; QoL = quality of life.

## Data Availability

The data presented in this study are available on request from the corresponding author. The data are not publicly available due to patient confidentiality.
